# A Simplified Direct Lipid Mixing Lipoplex Preparation: Comparison of Liposomal-, Dimethylsulfoxide-, and Ethanol-Based Methods

**DOI:** 10.1038/srep27662

**Published:** 2016-06-21

**Authors:** Joseph W. Meisel, George W. Gokel

**Affiliations:** 1 Center for Nanoscience, University of Missouri – St. Louis, 1 University Blvd., St. Louis, MO 63121, USA; 2 Department of Chemistry & Biochemistry, University of Missouri – St. Louis, 1 University Blvd., St. Louis, MO 63121, USA; 3 Department of Biology, University of Missouri – St. Louis, 1 University Blvd., St. Louis, MO 63121, USA

## Abstract

Established transfection methodology often uses commercial reagents, which must be formed into liposomes in a sequence of about half a dozen steps. The simplified method reported here is a direct lipid mixing approach that requires fewer steps, less manipulation, and is less time-consuming. Results are comparable to those obtained with more commonly used methods, as judged by a variety of analytical techniques and by comparisons of transfection results. The method reported here may be applied to non-liposome-forming compounds, thereby greatly expanding the range of structures that can be tested for transfection ability.

The tetravalent polyamine spermine was among the first reagents successfully employed in the transfection of mammalian cells over fifty years ago^1^. Shortly thereafter, DEAE-dextran, protamine, and other cationic polymers were found to increase the genetic transforming activity of viral DNA and RNA on bacterial and mammalian cells^2^,^3^,^4^,^5^. A technique utilizing the co-precipitation of calcium phosphate with DNA marked one of the first chemical transfection methods not using polyamines^6^.

Since these earliest studies, numerous procedures have been developed for non-viral mammalian cell transfection. Several physical techniques for transfection have been explored. These include gene gun^7^, electrospray^8^, electroporation^9^, sonoporation^10^, and magnetofection^11^. Chemical approaches typically involve the use of cationic polymers^12^, calcium phosphate^6^, inorganic nanoparticles^13^, and lipids^14^,^15^.

The formation of stable and condensed particles containing DNA is the most commonly used technique for *in vitro* transfection. When such a complex is formed using a cationic polymer it is called a *polyplex*, whereas cationic lipids beget a *lipoplex*. While cationic polymers are typically water soluble, cationic lipids are not. The latter must therefore be pre-formed into liposomes^16^. The lipoplex is then generated by the addition of an aqueous solution of cationic liposomes to an aqueous solution of DNA. The spontaneously formed lipoplex transfects cells in the process known as *lipofection*^15^.

The lipoplex structure does not resemble that of the small unilamellar liposomes from which they derive. Instead, the lipoplex is a multilamellar liquid crystal consisting of hydrated DNA layers alternating with cationic lipid bilayers^17^. A structural variant consists of a columnar hexagonal phase in which the DNA helix comprises the axis. Cationic lipid head groups face the DNA and the hydrophobic tails interdigitate between the hexagonally packed columns excluding water^18^. The dynamics of lipoplex assembly are poorly understood, but it has been shown that lipid packing parameters^19^ dictate the structure’s organization.

In most reports involving transfection, lipoplex formation was predominantly achieved by mixing plasmid DNA with cationic liposomes formed by the well-established lipid film hydration method^20^. Small unilamellar vesicles are achieved by sonication and/or extrusion^21^ of the hydrated lipid. An ethanol-injection method has also been reported for liposome formation^22^ and used in transfection^23^. These and other lipoplex preparation methods based on a liposomal intermediate require the use of lipids or lipid mixtures that form stable liposomes. Unless an amphiphile can form liposomes, it cannot be tested for DNA interaction or transfection using these methods.

Lipoplex preparation methods have been developed that eliminate the liposomal intermediate. One method requires the use of a T-shaped mixing chamber to add an ethanolic solution of lipids to aqueous DNA^24^. Another method utilizes 50% aqueous ethanol to dissolve both lipids and DNA and the resulting particles have been called *Genospheres*^TM ^25. Both methods require rotary evaporation or dialysis to remove ethanol before lipoplex delivery to cells.

The results presented here demonstrate that lipids dissolved in dimethylsulfoxide (DMSO) or ethanol (EtOH) may be added directly to aqueous DNA to form a lipoplex. We call this technique *direct lipid mixing* (DLM) and denote the solvent by subscript, *i.e.* DLM_DMSO_ or DLM_EtOH_. This method is fast, simple, and requires neither specialized equipment nor removal of the organic solvent. The method enables facile optimization of multi-lipid mixtures and lipid-DNA ratios. The absence of a liposomal intermediate enables the investigation of a greater diversity of chemical structures for transfection ability.

## Results and Discussion

### Model cationic lipid transfection agent

**Figure 1:**
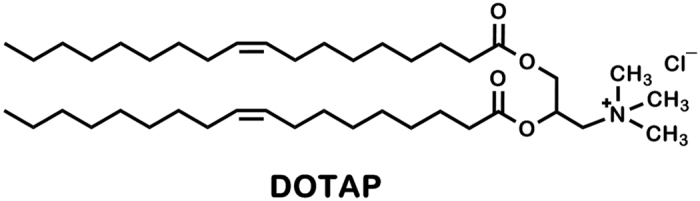
Chemical structure of model cationic lipid transfection reagent 1,2-dioleoyl-3-trimethylammoniumpropyl chloride (DOTAP).

The studies presented here use the cationic lipid 1,2-dioleoyl-3-trimethylammoniumpropyl chloride (DOTAP, see Fig. 1). This lipid is widely used as an *in vitro* transfection agent^26^,^27^, its lipoplex with DNA has been characterized^18^,^28^, and it is commercially available. Liposomes can easily be prepared from DOTAP alone without the need for a helper lipid (such as dioleoylphosphatidylethanolamine, DOPE). DOTAP is also soluble in the water-miscible organic solvents DMSO and ethanol. This property makes it possible to assess if DOTAP functions differently in lipoplex formation depending on whether the lipid is initially isotropically dispersed in a solvent (DLM) or the lipid is in the form of aqueous liposomes. Such differences in lipoplex formation could affect DOTAP’s activity as a transfection agent. It is this question that is addressed herein.

### Assessment of DOTAP-DNA interaction by agarose gel electrophoresis

DNA migrates through an agarose gel matrix by the action of an electric field according to its charge, length, and morphology. Successful transfection reagents, whether polymers or lipids, inhibit the migration of plasmid DNA through the gel. This retardation may result from aggregate formation, which screens the phosphates from the influence of the electric field.

We compared the effects of concentration and the physical state of DOTAP on the migration of plasmid DNA in an agarose gel in order to assess whether DLM or liposomal methods influence the DNA-lipid interaction. Lipoplex solutions were formed either by the addition of aqueous liposomal DOTAP or DMSO-dissolved DOTAP (DLM_DMSO_) to an aqueous solution of 10 kilobase-pair (10 kb) plasmid DNA. Aqueous liposomes were prepared by two methods: by sonication or sonication-extrusion (hereafter referred to only as “extrusion”). Lipoplex solutions of DOTAP:DNA phosphate (+/− charge) ratios from 0.25 to 4 were prepared and added to the wells of an agarose gel. We were unable to test ethanol-dissolved DOTAP (DLM_EtOH_) because the mixture is less dense than water. This precludes the loading of the mixture into the wells. The electrophoretic mobility of the DNA was observed by ethidium bromide staining.

**Figure 2:**
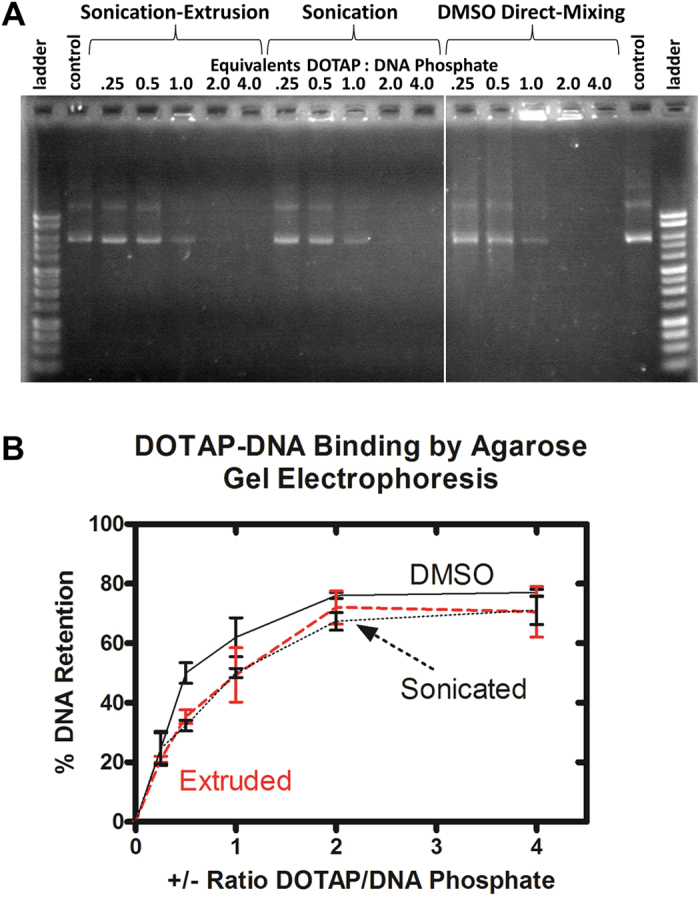
(**A**) Agarose gel electrophoresis of 10 kb plasmid DNA-DOTAP lipoplexes formed with liposomes (sonicated or extruded) or by DLM_DMSO_. The image represents a single gel; the gap in the image omits a control plasmid lane for clarity. (**B**) Percent retention of DNA by DOTAP as a function of +/− charge ratio and initial lipid form calculated by densitometric transformation of agarose gel images. Error bars represent the standard deviation of three trials.

We found that the DNA-lipid interaction as assessed by electrophoretic mobility was independent of the initial lipid physical state. The gel image in Fig. 2A shows that both liposomal and DLM_DMSO_ DOTAP interact with DNA to the same extent. At DOTAP:DNA phosphate ratios of 1–2, nearly all of the DNA was retained independent of the initial lipid physical state. Substoichiometric amounts of cationic lipid resulted in some DNA retention. The absence of significant streaking suggested the presence of minor amounts of intermediate structures, *i.e.* partially formed lipoplexes. The latter result suggested that lipoplex formation may be cooperative.

The graph of Fig. 2B confirms that the concentration-retention profile for each lipid form is nearly identical. Together, these data suggest that the primary function of preparing a liposomal form of lipofection reagents is to make the lipids water-soluble. Once the liposomes and DNA interact, the liposomal structure collapses and the lipids and DNA co-assemble into a distinct lipoplex structure. By dissolving the lipid in DMSO or ethanol, the liposome preparation step can be eliminated.

The stability of transfection reagents is an important consideration when assessing the convenience of methods. Liposomes are often stored at 4 °C with a shelf life from weeks to months with many factors contributing to their stability^29^. The chemical degradation of phospholipids by oxidation or hydrolysis and the physical stability of liposomes in suspension can be responsible for lower transfection efficiency or higher toxicity. The DLM method possesses major advantages relevant to stability. First, DLM lipid solutions may be prepared immediately prior to use much more rapidly than liposome solutions. Second, because DLM lipid solutions are isotropically dispersed they are not susceptible to the physical stability (*i.e.* aggregation) issues encountered with liposomes. Finally, DLM_DMSO_ solutions may be particularly stable as DMSO readily freezes at 4 °C. In order to assess stability, the DNA retention of sonicated, extruded, and DLM_DMSO_ solutions stored at 4 °C for 6 months was tested by electrophoresis and found no difference in DNA mobility was apparent when compared to fresh solutions (see supporting information).

### Lipoplex characterization by dynamic light scattering (DLS)

Both liposomes and lipoplex particles were studied in aqueous solution by dynamic light scattering. DOTAP liposomes were prepared by sonication or extrusion and diluted to a concentration of 30 μM in water. DLS showed that the average particle size was 350 nm for the sonicated lipid, whereas extrusion resulted in 180 nm particles (corresponding well to the 0.2 μm (200 nm) membrane filter pore). The size distribution for liposomes formed by sonication alone was broader than for liposomes formed by extrusion. When DMSO-dissolved lipids were diluted with water to 30 μM, 90 nm particles were observed and fewer particles were apparent than was recorded for either liposomal method. In the latter case, some lipids may be dispersed as monomers or aggregates that were too small to be detected.

A so-called “simple injection” technique for liposome formation that was reported some years ago involves ethanol-dissolved lipids^23^. A DLS analysis of particle sizes obtained by using this technique found too few particles to measure. Presumably, the lipids were mostly dispersed as monomers or perhaps as aggregates too small to be detected. Based on the specifications of the instrument used in our analyses, the former seems more likely. The simple-injection procedure requires rapid injection of ethanol-dispersed lipids into water with simultaneous vortexing to form liposomes. Notwithstanding its potential convenience, this method has been used only occasionally in transfection studies during the past two decades.

The charge affinity between the cationic lipids and the DNA phosphate groups drives the self-assembly. It is also likely that solvent exclusion from the complex also fosters its formation. DLS showed that directly mixing DMSO-dissolved lipids with DNA (DLM_DMSO_) resulted in monodisperse lipoplex structures.

**Figure 3:**
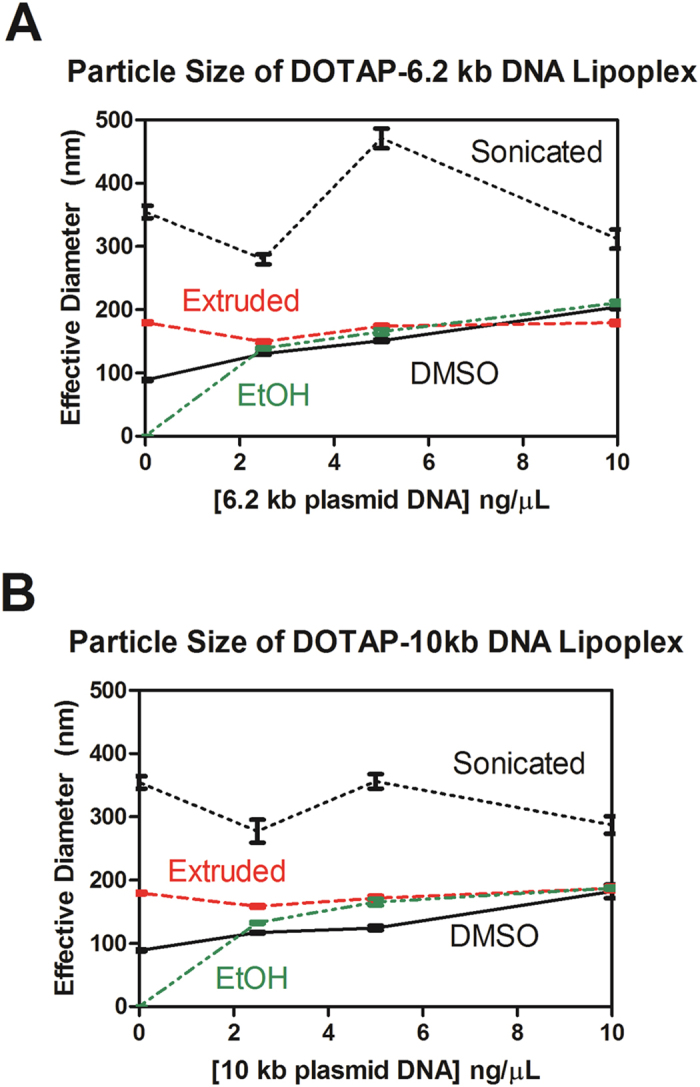
Assessment of particle size variations for 30 μM DOTAP with varying amounts of plasmid DNA. Sonicated and extruded DOTAP are liposomal methods. EtOH and DMSO DOTAP are direct lipid mixing methods. Data shown represent the average of 10 measurements.

Figure 3 shows particle size (DLS) data for lipoplexes formed by each of the four methods discussed here. They are (1) sonication, (2) extrusion, and the present method using (3) DMSO or (4) ethanol as the lipid solvent. Lipoplexes were formed using DOTAP and 6.2 kb or 10 kb plasmid DNA. The results demonstrate that methods 2–4 afford lipoplex particles that are similar in size. However, aqueous lipids that were sonicated formed larger liposomes. Comparing the results of similar experiments conducted with 6.2 kb and 10 kb plasmids show that the lipoplex reflects the initial liposome size rather than the length of the DNA included within it.

The size range of lipoplexes measured was relatively monodisperse across all preparation methods. The polydispersity index (PDI) was less than 0.25 for all methods with sonication giving the widest size distribution (PDI ~0.25). DLM methods had a typical PDI of 0.15. Unsurprisingly, extruded liposomes produced the most uniform lipoplexes, which had a PDI of ~0.1. Under our preparation conditions, both the size and polydispersity of lipoplexes were reproducible in repeated trials.

### Transmission electron microscopy of DOTAP-DNA lipoplex

The size and morphology of DOTAP-DNA lipoplexes prepared by extrusion or by DLM_DMSO_ were examined by using transmission electron microscopy. Lipoplexes were adhered to formvar/carbon-coated copper grids and negatively stained with uranyl acetate. Both extrusion and DLM_DMSO_ methods resulted in multilamellar structures. Lamellar spacings in extrusion samples were 4.7 ± 1.2 nm. A similar lamellar spacing (4.6 ± 0.5 nm) was observed in samples prepared by the DLM_DMSO_ method. These spacings correspond to lamellae consisting of DNA alternating with DOTAP bilayers.

We clearly observe lamellar structures for the lipoplexes formed by using the DLM_DMSO_ method. The lamellar spacing is approximately 5 nm (50 Å). Lamellar spacings recorded in other lipoplex formation reports are larger than this, but reflect different experimental conditions. Talmon and coworkers reported a spacing of 4.9 nm for DOTAP with single-stranded oligonucleotides measured by cryo-TEM and by small angle X-ray scattering (SAXS)^30^. Safinya and coworkers reported lamellar spacings of 3.72 nm (37.2 Å) for DOTAP in the absence of DNA^31^. This finding implies lipid tail interdigitation, as our estimation of the length of a DOTAP monomer is 2.6 nm (26 Å). In the present case, the lipoplexes are supported (formvar backing) and are neither in solution nor suspension. If we estimate the DNA cylindrical diameter to be 2 nm (20 Å), the lipids must occupy ~3 nm (30 Å). The repeating unit must therefore include 20 Å + 15 Å + 15 Å to account for the spacing of 50 Å. This seems possible only if the lipids are interdigitated. Such compression seems reasonable because the solid TEM sample is unlikely to be hydrated.

**Figure 4:**
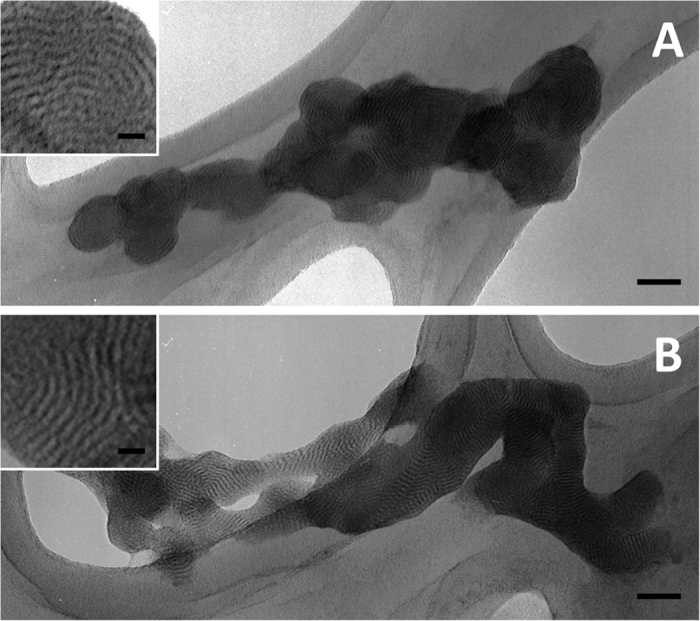
(**A**) Aggregated lipoplex particles formed from extruded DOTAP liposomes (**B**) Aggregated and partially-fused lipoplex particles formed from DMSO-dissolved DOTAP. The lamellar spacing is approximately 5 nm (50 Å) and is shown in the magnified inset. Scale bars represent 50 nm, inset scale bars represent 10 nm.

Despite the similarity in lamellar spacing, extrusion lipoplexes maintain a concentric lamellar structure (see Fig. 4A), whereas DLM_DMSO_ lipoplexes have a stacked lamellar arrangement (Fig. 4B). It is unclear whether the lipoplex preparation method or the TEM grid preparation accounts for the difference in morphology. We speculate that lipoplex formation from liposomes may be templated by the liposome, resulting in the curvature observed in the concentric lamellae. However, lipoplex formation from dissolved lipids may be templated by the DNA or by small lipid assemblies. The latter could result in a lipoplex lamellar structure that is influenced by the lipid packing parameters. This comports with DOTAP (a low-curvature lamellae-forming lipid) forming the stacked lamellar structure. Interestingly, Lehn and coworkers observed stacked lamellar structures for lipoplexes derived from a water-soluble guanidinium-cholesterol reagent (BGTC) by cryo-TEM^32^. However, when BGTC was formulated with DOPE as a liposome, concentric lamellar structures were observed for the corresponding lipoplex. This comports with our speculation above on liposome-templation versus DNA-templation or small lipid assembly-templation.

### Transfection using DMSO-dissolved lipids

**Figure 5:**
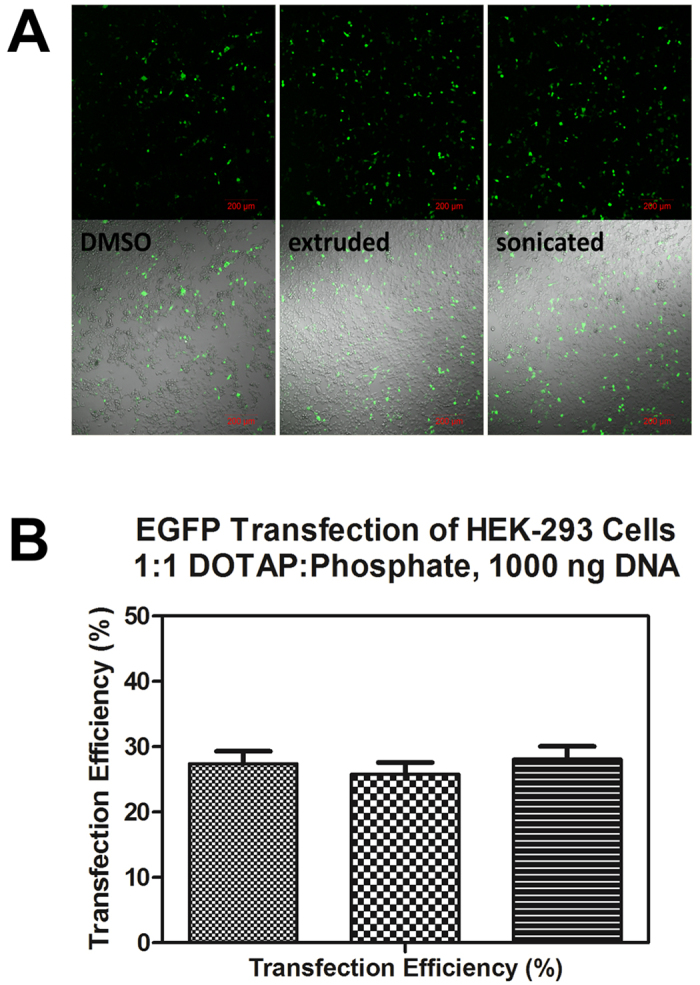
(**A**) Confocal micrographs of DOTAP-transfected HEK-293 cells using DMSO-solvated DOTAP (left), aqueous extruded liposomal DOTAP (middle), and aqueous sonicated liposomal DOTAP (right). The top darkfield images show EGFP fluorescence alone, bottom images are of the same cells with the fluorescence channel overlayed with brightfield to show cell density. (**B**) Transfection efficiency as determined by cell counting, expressed as a percentage of transfected cells. Error bars represent a ±7% error associated with cell counting.

**Figure 6:**
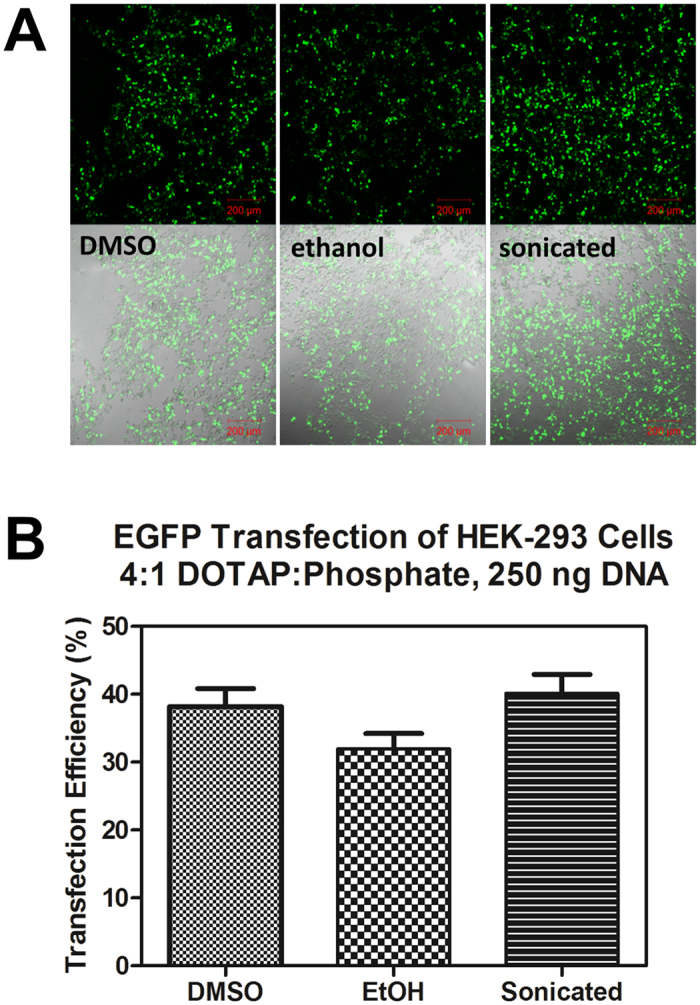
(**A**) Confocal micrographs of DOTAP-transfected HEK-293 cells using DMSO-solvated DOTAP (left), ethanol-solvated DOTAP (middle), and aqueous sonicated liposomal DOTAP (right). The top darkfield images show EGFP fluorescence alone, bottom images are of the same cells with the fluorescence channel overlayed with brightfield to show cell density. (**B**) Transfection efficiency as determined by cell counting, expressed as a percentage of transfected cells. Error bars represent a ±7% error associated with cell counting.

An application study was undertaken to determine if the similarities in lipoplex structures on electrophoretic, light scattering, and electron microscopic evidence were reflected in cells. Transfection studies were conducted on HEK-293 human embryonic kidney cells. The 6.2 kb plasmid pCDNA3-EGFP encoding the enhanced green fluorescent protein (EGFP) was used to visually identify transfected cells. Confocal microscopy was used to assay transfection efficiency. Transfection studies were run in triplicate and data was derived from representative confocal micrographs. Micrographs were analyzed using ImageJ software for cell counting (~2,500 cells per sample). Hand counting and computer analysis agreed within ±7%, represented by error bars in Figs 5B and 6B.

Both the DLM_DMSO_ and liposomal methods resulted in transfected cells. The first transfection study used 1000 ng of DNA per well and lipoplexes were formed from a 1:1 +/− ratio of DOTAP to DNA phosphate. The amount of DNA and DOTAP lipid used in each experiment was identical and is unoptimized for this cell line.

Figure 5 shows the results of this study, which compared both liposomal methods and the DLM_DMSO_ transfection method. The micrographs shown qualitatively demonstrate comparable transfection rates between methods. Quantitative analysis by cell-counting corrects for variable cell density and confirms that transfection efficiencies are similar.

In order to confirm that similar transfection efficiency was observed under different conditions, we performed an additional transfection at a lower DNA concentration (250 ng per well) and a greater DOTAP:DNA ratio (4:1). The amount of DOTAP per well was the same as in the previous experiment. Lipoplexes in this experiment were prepared by DLM_EtOH_ as well as the previously studied DLM_DMSO_ and sonicated liposome methods. The results from this study are shown in Fig. 6. Transfected cells were observed for each lipoplex preparation method. The rates of transfection for this DNA and DOTAP concentration regime were uniformly higher than in the previous transfection. The transfection efficiency was similar across all lipid formulation methods. These data suggest that DLM methods and liposomal methods give comparable transfection results under varied transfection conditions.

**Figure 7:**
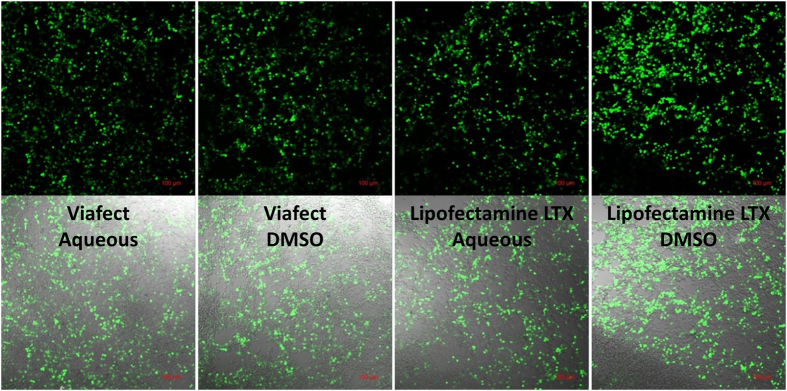
Confocal microscopy images of EGFP transfected cells. Top row is darkfield and bottom is brightfield image. Transfection was performed using the commercial reagent Viafect^TM^ as an aqueous liposomal solution (far left) and as a DMSO solution (middle left) and the commercial transfection reagent Lipofectamine LTX^TM^ as an aqueous liposomal solution (middle right) and as a DMSO solution (far right).

After obtaining transfection results for the direct-mixing lipoplex formation method with model lipid DOTAP, we examined its applicability to commercial lipofection reagents. The Lipofectamine^TM^ reagents have been used to transfect a variety of cell lines. Viafect^TM^ is a relatively new formulation that claims low cell toxicity. The exact formulations of the reagents are proprietary, but both are aqueous lipid-based solutions. A known volume of the commercial transfection reagent was dried by lyophilization and reconstituted in the same volume of DMSO. This dissolves the liposome assembly, leading to an isotropic solution. The DMSO-dissolved lipids were then mixed with DNA in the same ratio as the aqueous solutions and the resulting lipoplexes were used to transfect HEK-293 cells. Confocal micrographs of the transfected cells are displayed in Fig. 7. A quantitative determination of transfection efficiency or fluorescence intensity was not possible in this study due to high cell density. The DNA amount and lipid ratio have not been optimized for this cell line, but in each case both the aqueous and DLM_DMSO_ reagents successfully transfected the cells.

### Transfection and toxicity assessed by flow cytometry

**Figure 8:**
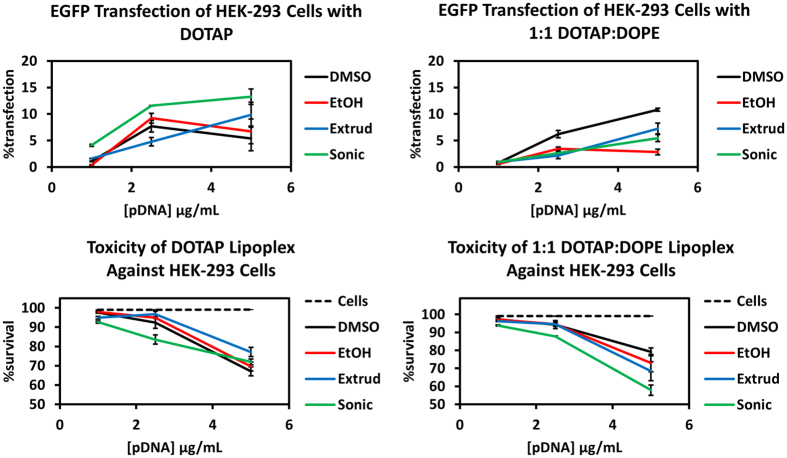
Transfection (top) and toxicity (bottom) of DLM and liposomal methods in human embryonic kidney cells. Single-lipid (left) and binary lipid (right) mixtures were assayed.

In order to demonstrate the utility and versatility of the direct lipid mixing method we performed additional transfection experiments using flow cytometry to determine transfection efficiency and toxicity. Transfection efficiency was assessed by EGFP fluorescence and toxicity was determined using the cell impermeant dye propidium iodide (PI)^33^. For the widespread applicability of this method, it must be useful with multicomponent lipid mixtures in addition to single lipid formulations. The top panel of Fig. 8 shows the results of the transfection of HEK-293 cells with lipoplexes derived from DOTAP (left) or from 1:1 DOTAP:DOPE (right). In each case the molar ratio of total lipid to DNA phosphate was 4:1. Three DNA concentrations were tested: 1.0, 2.5, and 5.0 μg/mL. The associated toxicities of these methods are represented in the bottom panel of Fig. 8. In each case, the toxicity of the DLM methods is similar to or less than that observed by using the liposomal methods. The transfection efficiency of DLM methods with DOTAP were similar to liposomal methods at 2.5 μg/mL DNA, but slightly lower at 5.0 μg/mL. The toxicity of DLM methods was less than the liposomal methods when cationic lipids were combined with a helper lipid DOPE. In transfection with the binary lipid system, DLM_EtOH_ was less toxic under these conditions but resulted in low transfection efficiency at 5 μg/mL DNA. DLM_DMSO_ performed significantly better than all other methods having both the highest transfection efficiency and lowest toxicity.

**Figure 9:**
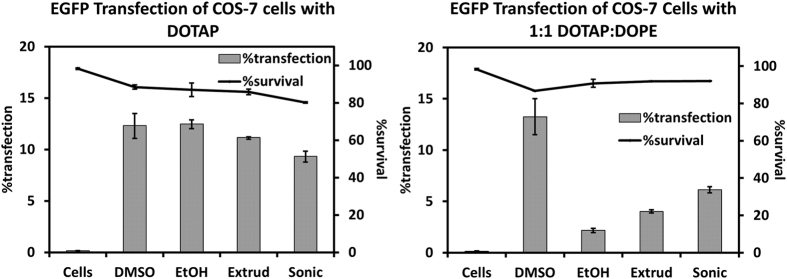
Transfection (bars) and toxicity (lines) of DLM and liposomal methods in COS-7 monkey kidney cells. Single-lipid (left) and binary lipid (right) mixtures were assayed.

In addition to assessing transfection efficiency and toxicity for DLM and liposomal methods with different lipid mixtures and various DNA concentrations, the application of DLM in a different cell line was also tested. The COS-7 cell line was chosen because of its widespread use in transfection for the preparation of recombinant proteins^34^. The transfection was carried out at 5.0 μg/mL DNA, 4:1 total lipid to DNA phosphate, and with DOTAP or DOTAP:DOPE lipid mixtures. The results are shown in Fig. 9.

The transfection of COS-7 (monkey kidney) cells by DLM or liposomal methods with DOTAP or DOTAP:DOPE lipoplexes at 5.0 μg/mL DNA showed generally lower toxicity than the same transfection conditions in HEK-293 cells. When DOTAP was used alone, DLM and liposomal methods transfected COS-7 cells with similar efficiency and toxicity. For the binary lipid system the transfection results were similar to those found with HEK cells. DLM_DMSO_ was the most efficient, liposomal methods were intermediate, and DLM_EtOH_ was the least effective. The appearance of this trend across two cell lines suggests that DMSO is the preferred solvent for direct lipid mixing for multi-lipid systems at higher DNA concentrations.

The use of DMSO in this procedure deserves comment. Since this solvent’s introduction into biological studies, the reports of its effects have varied from extremely favorable in certain applications to those uses where its presence was inimical to the desired outcome. We recently showed that the application of DMSO in a series of related systems showed some variation in outcome under similar conditions^35^. Notwithstanding, the studies presented here show that when used as prescribed, the transfection outcomes are reproducible and comparable to procedures that require greater manipulation.

The primary utility of this method, and indeed the conditions under which it has been tested, is for *in vitro* transfection with plasmid DNA. The application of the method to *in vitro* oligonucleotide delivery is under investigation. While not currently being tested, the extension of this method to *in vivo* transfection studies is not precluded. Organic solvents have long been used safely to solubilize drugs for parenteral applications and as embolic liquids themselves^36^. While the initial mixing of lipid and DNA in the DLM method results in a solution of 15–50% by volume of organic solvent, the solution delivered to cells consisted of only 1% organic solvent or less and was effective in media with or without serum. DMSO is listed as an inactive ingredient in approved drugs in the United States for topical administration in three FDA applications and for a single intravenous application (lyophilized powder, DMSO content not specified)^37^. Ethanol is listed as an inactive ingredient in many topical applications and at least 15 intravenous or intramuscular applications with intravenous injection concentrations as high as 92%.

## Conclusion

**Figure 10:**
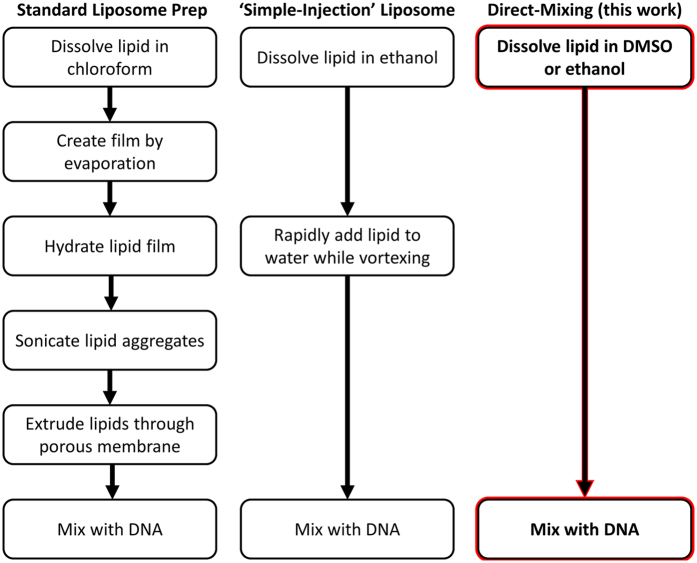
Flow chart demonstrating lipoplex methods compared herein. Depicted are the liposomal methods of standard lipid film hydration-sonication-extrusion (left), simple-injection method (middle), and the non-liposomal direct lipid mixing (DLM) method featured in this work (right).

The present approach is a simplification of established methodology. It uses known lipids to form lipoplexes that are successfully transfected into mammalian cells. The studies presented here are side-by-side comparisons of two previous methods with the present approach. The key advantages of the method described here are convenience, cost, and efficiency. The comparative data confirm that the present approach affords transfection results that are comparable to those obtained by using methods that are in more common use. A summary diagram comparing the known and present methods is shown as Fig. 10.

Taken together, the results reported in this work demonstrate that the liposomal structure itself is not a necessary prerequisite to lipoplex formation. Liposome formation can be bypassed by dissolving lipids in an aqueous-miscible organic solvent and mixing directly with an aqueous solution of DNA. The resulting lipoplexes transfected HEK-293 and COS-7 mammalian cells with efficiencies comparable to the standard protocol. This was demonstrated with the model lipid DOTAP dissolved either in dimethylsulfoxide or in ethanol. Our results suggest that the DLM_DMSO_ method is advantageous in binary or multi-lipid systems. We also provide evidence that the DLM_DMSO_ method works with the commercial lipid formulations Lipofectamine LTX^TM^ and Viafect^TM^.

By obviating the necessity for liposome-forming lipids, this work greatly expands the range of chemical structures that can be tested for transfection ability. Furthermore, the process of optimizing lipid formulations to a particular cell line is simplified. Of course, the choice of solubilizing solvent is an important consideration. While DMSO and ethanol were the solvents tested in this study, other solvents might be used. Minimally, the solvent must dissolve the transfection reagents and be miscible with water. The biological effects of such solvents on their own should also be examined and controlled for, with our results indicating that DMSO and ethanol have no observable effect on toxicity at the concentrations used. We anticipate that the development of the DLM method, while modest, may potentiate the pursuit of higher efficiency, more selective transfection reagents for biological research and gene therapy.

## Materials and Methods

Lipofectamine^TM^ and Viafect^TM^ were obtained from commercial sources as aqueous suspensions. DOTAP was obtained from Avanti Polar Lipids (Alabaster, AL, USA) in solid form as the chloride salt. Plasmid DNA (pKLMF-FX, 9.988 kb, New England Biolabs or pCDNA3-EGFP, 6.160 kb, Addgene # 13031) was amplified in *E. coli,* extracted using Zyppy^TM^ Maxiprep spin columns, purified by NaCl/ethanol precipitation, and dissolved in 18.2 MΩ purified water.

### Lipid preparations

#### Sonicated and extruded DOTAP liposomes

A 1.0 mL solution of 6.06 mM (or 24.24 mM for transfection) DOTAP-Cl in chloroform was prepared in a clean glass vial. Chloroform was removed by rotary evaporation to leave a thin film of lipid. The lipid film was hydrated with 1.0 mL 18.2 MΩ purified water, vortexed, and sonicated to homogeneity. Extruded liposomes were prepared from sonicated lipids by passing the solution through a 0.2 μm Whatman filter membrane 11 times.

#### DMSO (or ethanol)-dissolved DOTAP lipid

A 1.0 mL solution of 6.06 mM (24.24 mM for transfection) DOTAP-Cl in dimethyl sulfoxide (or ethanol) was prepared in a clean glass vial. The solution was incubated at 37 °C on an orbital shaker at 50 rpm and vortexed to ensure complete dissolution.

#### DMSO-Dissolved commercial transfection solutions

A 100 μL solution of Lipofectamine LTX^TM^ or Viafect^TM^ formulation was lyophilized on a Labconco Lyph Lock 6 freeze dryer. The residue was then dissolved in 100 μL DMSO and used as directed.

### Transfection procedure

Transfection was carried out on HEK-293 cells in antibiotic-free media on black Nunc^TM^ 96-well optical-bottom plates (Thermo Scientific). Plates were seeded with 20,000 cells/well in antibiotic-free Dulbecco’s modified Eagle’s medium (DMEM), 10% fetal bovine serum (FBS) and grown to approximately 70% confluency before transfection. DOTAP-pCDNA3-EGFP lipoplexes of 1:1 +/− ratio were prepared by mixing 5 μL 6.06 mM DOTAP (sonicated, extruded, DMSO-dissolved, or EtOH-dissolved) with 100 μL of 100 ng/μL plasmid DNA. Lipoplex-containing media was prepared by adding 42 μL lipoplex solution to 400 μL DMEM (FBS-free). Lipoplexes of 4:1 +/− ratio were formed by the same procedure using 24.24 μM lipid and diluted 4-fold with 18.2 MΩ H_2_O, controlling for DMSO or ethanol content for DLM methods. Cell media was removed and 110.5 μL lipoplex media was added to cells and incubated 90 min, 37 °C, 5% CO_2_ before 200 μL DMEM (containing blasticidin and 10% FBS) was added. Cells received either 1000 ng DNA at 1:1 +/− ratio or 250 ng DNA at 4:1 +/− ratio. The final concentration of DMSO or ethanol was less than 0.5%. After 36 h, cell media was replaced with phosphate-buffered saline and cells were imaged on a Zeiss LSM 700 confocal microscope.

### Flow Cytometry

Transfection for flow cytometry experiments was performed in 24-well plates treated for cell culture. Cells were seeded in 1 mL aliquots at 50,000 cells per well in DMEM with 10% FBS and grown to about 70% confluency overnight. Lipoplexes were prepared as described above for a 4:1 total lipid to DNA phosphate molar ratio. DOTAP trials were performed in triplicate and DOTAP:DOPE trials were performed in duplicate. Transfection was performed in 0.5 mL DMEM (FBS-free) containing the lipoplex for 90 min, 37 °C, 5% CO_2_ before 0.5 mL DMEM (10% FBS) was added. The DNA concentration before the DMEM + FBS supplement for HEK-293 transfection was 1.0, 2.5, or 5.0 μg/mL; COS-7 cells were transfected in an identical manner at 5.0 μg/mL DNA. After 24 h, the cell media was removed and centrifuged at 100 × g for 5 min to collect dead cells (for toxicity assay) and the supernatant was removed. Adherent cells were collected by washing with 0.3 mL PBS, adding 0.3 mL 0.25% trypsin-EDTA solution and incubating 5 min at 37 °C, then 0.7 mL DMEM + FBS was added and the cells were combined with dead cells and centrifuged at 100 × g for 10 min. The supernatant was removed and cells were resuspended in 300 μL DMEM + FBS. To stain dead cells 0.5 μL 0.5 mg/mL propidium iodide (PI) solution was added. Cells were analyzed on a BD Biosciences FACSCanto II. EGFP was detected by excitation at 488 nm using a 530/30 nm filter, 520 LP mirror; PI was detected by excitation at 488 nm using a 670 nm filter, 655 LP mirror. Toxicity and transfection efficiency were determined by gating out cell debris and establishing a quadrant analysis based on PI +/− and EGFP +/−. Toxicity is reported as %survival = (PI-/EGFP- + PI-/EGFP+)/total cells. Transfection efficiency is reported as %transfection = (PI-/EGFP+)/total cells. After gating out debris each analysis contained about 30,000 cells on average.

### Agarose Gel Electrophoresis

Agarose powder was obtained from Sigma Aldrich. Purified water with 18.2 MΩ resistivity (Milli-Q) was used in all cases. Gels were cast by heating a 0.5% w/v solution of agarose in 40 mM tris-acetate buffer (pH 7.2) until fully dissolved, then cast by cooling the solution to room temperature in an Owl B2 horizontal electrophoresis chamber with a centrally placed 20-well comb. The gel was submerged under 40 mM tris-acetate (pH 7.2) running buffer, samples were added to the wells, and the gel was run 90 minutes at 105 ± 3 volts. Gels were stained in 2.5 μg/mL ethidium bromide for 15 minutes at 37 °C, 50 rpm, and destained in Milli-Q water for 5 minutes at 37 °C, 50 rpm. Ethidium bromide-stained DNA was visualized using a UV trans-illuminator.

Gel images were analyzed by densitometry using ImageJ software^38^. Lane profile plots were generated and integrated (data not shown). Using manual baselines, the control plasmid was set as 100% DNA migration. The inverse of the DNA migration in experimental wells relative to control DNA migration gave a measure of percent DNA retention. Results presented are the average of three gels.

### Dynamic Light Scattering

Measurements were performed on a Brookhaven Instruments Corp. ZetaPALS instrument at 37 °C using a 660 nm laser and correlating scattering at 90°. Samples were prepared by adding 10 μL 6.06 mM lipid solution to 200 μL of 25, 50, or 100 ng/μL pKLMF-FX or pCDNA3-EGFP in a pre-cleaned glass vial. The mixture was vortexed and incubated at room temperature for 5 minutes then diluted to 2.0 mL with 37 °C 18.2 MΩ H_2_O, transferred to a clean glass cuvette and equilibrated in the instrument for 5 min at 37 °C. Ten measurements consisting of two-minute runs were made on each sample. The average effective diameter was calculated with the standard deviation reported as the error.

### Transmission Electron Microscopy

The samples from dynamic light scattering measurements were also used for TEM. A 10 μL sample was applied to lacey formvar/carbon 300 mesh copper TEM grids (Ted Pella, Inc.) for 60 seconds and the grid was washed with 18.2 MΩ H_2_O (30 s), stained with 2% uranyl acetate (30 s), and washed twice with H_2_O (15 s each). The above solutions were applied at a volume of 10 μL, wicked away between each application, and the grid was finally dried with a gentle N_2_ stream. Specimens were examined on a JEOL JEM-2000 FX transmission electron microscope operated at 300 keV. Lamellae measurements were performed by ImageJ analysis of electron micrographs. Briefly, density profiles perpendicular to lamellae were generated from a minimum of 6 lipoplex structures per sample. Lamellar spacings were obtained by taking the first derivative of the density profile function.

## Additional Information

**How to cite this article**: Meisel, J. W. and Gokel, G. W. A Simplified Direct Lipid Mixing Lipoplex Preparation: Comparison of Liposomal-, Dimethylsulfoxide-, and Ethanol-Based Methods. *Sci. Rep.* **6**, 27662; doi: 10.1038/srep27662 (2016).

## Supplementary Material

Supplementary Information

## References

[b1] Szybalska E. H. & Szybalski W. Genetics of human cell lines, IV. DNA-mediated heritable transformation of a biochemical trait. Proc. Natl. Acad. Sci. USA 48, 2026–2034 (1962). 13980043 10.1073/pnas.48.12.2026 PMC221117

[b2] Vaheri A. & Pagano J.S. Infectious poliovirus RNA: a sensitive method of assay. Virology 27, 434–436 (1965). 4285107 10.1016/0042-6822(65)90126-1

[b3] McCutchan J. H. & Pagano J. S. Enhancement of the infectivity of Simian Virus 40 deoxyribonucleic acid with diethylaminoethyl-dextran. JNCI J. Natl. Canc. Institut. 41, 351–357 (1968). 4299537

[b4] Benzinger R., Kleber I. & Huskey R. Transfection of Escherichia coli spheroplasts I. general facilitation of double-stranded deoxyribonucleic acid infectivity by protamine sulfate. J. Virol. 7, 646–650 (1971). 4997550 10.1128/jvi.7.5.646-650.1971 PMC356175

[b5] Henner W.D., Kleber I. & Benzinger R. Transfection of Escherichia coli spheroplasts III. facilitation of transfection and stabilization of spheroplasts by different basic polymers. J. Virol. 12, 741–747 (1973). 4591047 10.1128/jvi.12.4.741-747.1973 PMC356692

[b6] Graham F.L. & van der Eb A.J. A new technique for the assay of infectivity of human adenovirus 5 DNA. Virology 52, 456–467 (1973). 4705382 10.1016/0042-6822(73)90341-3

[b7] Fynan E.F. *et al.* DNA vaccines: protective immunizations by parenteral, mucosal, and gene-gun inoculations. Proc. Natl. Acad. Sci. USA 90, 11478–11482 (1993). 8265577 10.1073/pnas.90.24.11478 PMC48007

[b8] Ikemoto K., Sakata I. & Sakai T. Collision of millimetre droplets induces DNA and protein transfection into cells. Sci. Rep. 2, 289 (2012). 22375250 10.1038/srep00289 PMC3289038

[b9] Neumann E., Schaefer-Ridder M., Wang Y. & Hofschneider P. H. Gene transfer into mouse lyoma cells by electroporation in high electric fields. EMBO J. 7, 841–845 (1982). 6329708 10.1002/j.1460-2075.1982.tb01257.x PMC553119

[b10] Marmottant P. & Hilgenfeldt S. Controlled vesicle deformation and lysis by single oscillating bubbles. Nature 423, 153–156 (2003). 12736680 10.1038/nature01613

[b11] Scherer F. *et al.* Magnetofection: enhancing and targeting gene delivery by magnetic force *in vitro* and *in vivo*. Gene Ther. 9, 102–109 (2002). 11857068 10.1038/sj.gt.3301624

[b12] De Smedt S. C., Demeester J. & Hennink W. E. Cationic polymer based gene delivery systems. Pharm. Res. 17, 113–126 (2000). 10751024 10.1023/a:1007548826495

[b13] Sokolova V. & Epple M. Inorganic nanoparticles as carriers of nucleic acids into cells. Angew. Chem. Int. Ed. 47, 1382–1395 (2008). 10.1002/anie.200703039 18098258

[b14] Fraleyt R., Subramani S., Berg P. & Papahadjopoulos D. Introduction of liposome-encapsulated SV40 DNA into cells. J. Biol. Chem. 255, 10431–10435 (1980). 6253474

[b15] Felgner P. L. *et al.* Lipofection: A highly efficient, lipid-mediated DNA-transfection procedure. Proc. Natl. Acad. Sci. USA 84, 7413–7417 (1987). 2823261 10.1073/pnas.84.21.7413 PMC299306

[b16] Li W. & Szoka F. C. Jr. Lipid-based nanoparticles for nucleic acid delivery. Pharm. Res. 24, 438–449 (2007). 17252188 10.1007/s11095-006-9180-5

[b17] Rädler J. O., Koltover I., Salditt T. & Safinya C. R. Structure of DNA–cationic liposome complexes: DNA intercalation in multilamellar membranes in distinct interhelical packing regimes. Science 275, 810–814 (1997). 9012343 10.1126/science.275.5301.810

[b18] Safinya C. Structures of lipid–DNA complexes: supramolecular assembly and gene delivery. Curr. Opin. Struct. Biol. 11, 440–448 (2001). 11495736 10.1016/s0959-440x(00)00230-x

[b19] Israelachvili J. N., Mitchell D. J. & Ninham B. W. Theory of self-sssembly of hydrocarbon amphiphiles into micelles and bilayers. J. Chem. Soc., Faraday Trans. 2 72, 1525–1568 (1976).

[b20] Bangham A. D., Standish M. M. & Watkins J. C. Diffusion of univalent ions across the lamellae of swollen phospholipids. J. Mol. Biol. 13, 238–252 (1965). 5859039 10.1016/s0022-2836(65)80093-6

[b21] Olson F., Hunt C. A., Szoka F. C., Vail W. J. & Papahadjopoulos D. Preparation of liposomes of defined size distribution by extrusion through polycarbonate membranes. Biochim. Biophys. Acta. 557, 9–23 (1979). 95096 10.1016/0005-2736(79)90085-3

[b22] Batzri S. & Korn E. D. Single bilayer liposomes prepared without sonication. Biochim. Biophys. Acta. 298, 1015–1019 (1973). 4738145 10.1016/0005-2736(73)90408-2

[b23] Campbell M. J. Lipofection reagents prepared by a simple ethanol injection technique. Biotechniques 18, 1027–1032 (1995). 7546703

[b24] Jeffs L. B. *et al.* A scalable, extrusion-free method for efficient liposomal encapsulation of plasmid DNA. Pharm. Res. 22, 362–372 (2005). 15835741 10.1007/s11095-004-1873-z

[b25] Hayes M.E. *et al.* Genospheres: self-assembling nucleic acid-lipid nanoparticles suitable for targeted gene delivery. Gene Ther. 13, 646–651 (2006). 16341056 10.1038/sj.gt.3302699

[b26] Stamatatos L., Leventis R., Zuckermann M. J. & Silvius J. R. Interactions of cationic lipid vesicles with negatively charged phospholipid vesicles and biological membranes. Biochemistry 27, 3917–3925 (1988). 3415963 10.1021/bi00411a005

[b27] Leventis R. & Silvius J. R. Interactions of mammalian cells with lipid dispersions containing novel metabolizable cationic amphiphiles. Biochim. Biophys. Acta 1023, 124–132 (1990). 2317491 10.1016/0005-2736(90)90017-i

[b28] Simberg D. *et al.* Phase behavior, DNA ordering, and size instability of cationic Lipoplexes. J. Biol. Chem. 276, 47453–47459 (2001). 11564736 10.1074/jbc.M105588200

[b29] Grit M. & Crommelin D.J. Chemical stability of liposomes: implications for their physical stability. Chem. Phys. Lipids 64, 3–18 (1993). 8242840 10.1016/0009-3084(93)90053-6

[b30] Weisman S., Hirsch-Lerner D., Barenholz Y. & Talmon Y. Nanostructure of cationic lipid-oligonucleotide complexes. Biophys. J. 87, 609–614 (2004). 15240493 10.1529/biophysj.103.033480 PMC1304382

[b31] Rädler J. O., Koltover I., Jamieson A., Salditt T. & Safinya C. R. Structure and interfacial aspects of self-assembled cationic lipid-DNA gene carrier complexes. Langmuir 14, 4272–4283 (1998).

[b32] Pitard B. *et al.* Structural characteristics of supramolecular assemblies formed by guanidinium-cholesterol reagents for gene transfection. Proc. Natl. Acad. Sci. USA 96, 2621–2626 (1999). 10077560 10.1073/pnas.96.6.2621 PMC15818

[b33] Petrunka A.M. & Harrison R.A.P. Mathematical analysis of mis-estimation of cell subsets in flow cytometry: viability staining revisited. J. Immunol. Methods 368, 71–79 (2011). 21362427 10.1016/j.jim.2011.02.009

[b34] Blasey H.D., Aubry J-P., Mazzei G.J. & Bernard A.R. Large scale transient expression with COS cells. Cytotechnology 18, 183–192 (1996). 22358744 10.1007/BF00767766

[b35] Negin S. *et al.* The aqueous medium-dimethyl sulfoxide conundrum in biological studies. RSC Adv. 5, 8088–8093 (2015).

[b36] Mottu F., Laurent A., Rüfenacht D.A. & Doelker E. Organic solvents for pharmaceutical parenterals and embolic liquids: a review of toxicity data. PDA J. Pharm. Sci. Technol. 54, 456–469 (2000). 11107838

[b37] United States Food and Drug Association Center for Drug Evaluation and Research, Inactive Ingredient Database, http://www.accessdata.fda.gov/scripts/cder/iig/index.cfm (Feb. 29, 2016), accessed April 30, 2016.

[b38] Rasband, W.S. ImageJ 1.47v. U. S. National Institutes of Health, Bethesda, Maryland, USA (1997–2015) Available at: http://imagej.nih.gov/ij/ (Accessed: 21st October 2015).

