# Supplementary material for: A Simplified Direct Lipid Mixing Lipoplex Preparation: Comparison of Liposomal-, Dimethylsulfoxide-, and Ethanol-Based Methods
Source: Sci Rep. 2016 Jun 21;6:27662. doi: 10.1038/srep27662 (PMC4914933; doi:10.1038/srep27662)
Supplement: Supplementary Information [file srep27662-s1.pdf]

Stability test of liposomes and lipids dissolved in DMSO

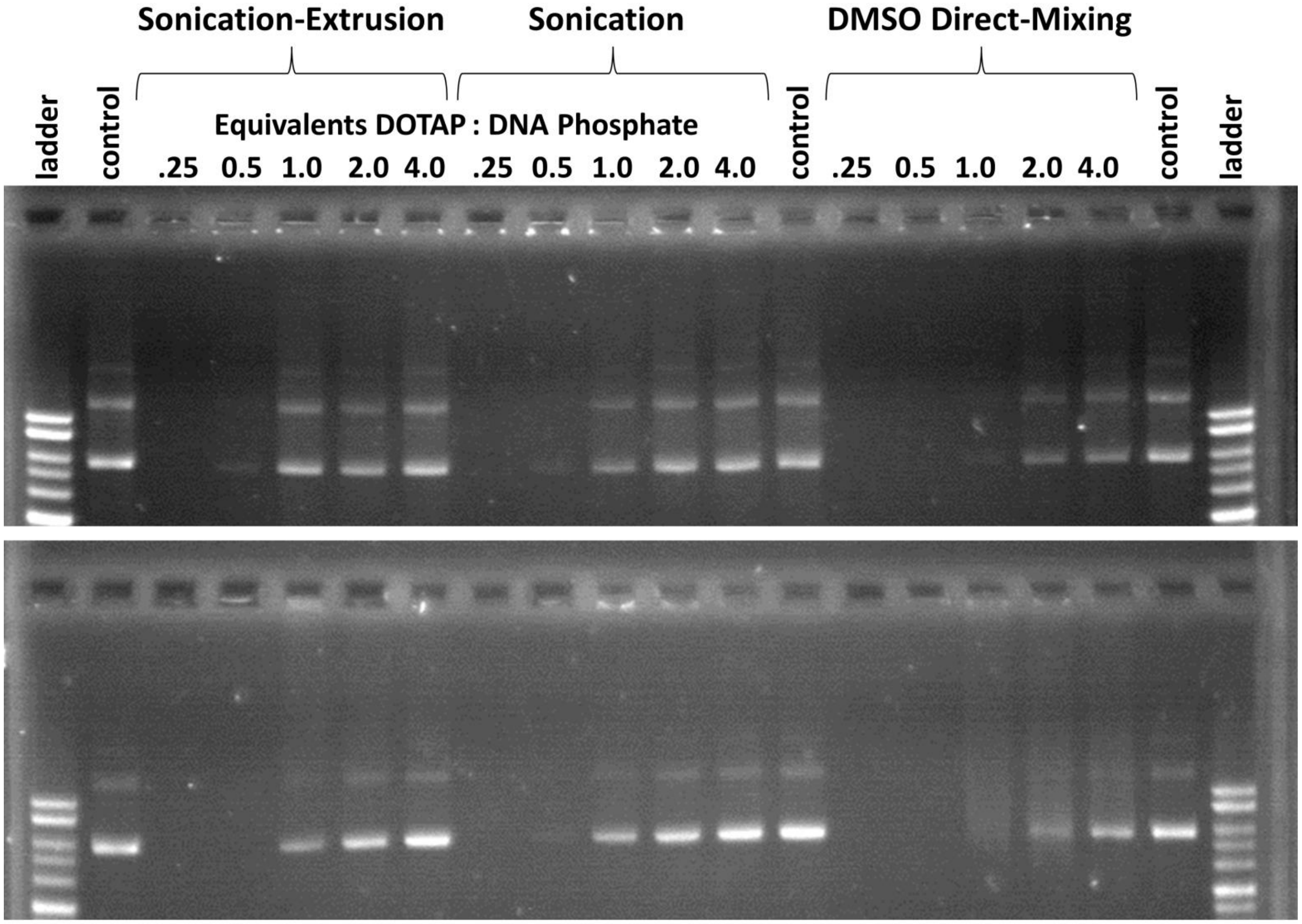

Bottom gel: lipids aged 6 months @ 4°C, no appreciable change
